# Social Determinants of Health and Health Care Seeking: Analysis of a Mexican Population-Based Survey

**DOI:** 10.1093/geroni/igaf122.2465

**Published:** 2025-12-31

**Authors:** Andrea Higareda-Garza-Ramos, Virgilio Hernández-Ruiz, José Ávila-Funes, Juan García-Lara

**Affiliations:** Instituto Nacional de Ciencias Médicas y Nutrición Salvador Zubirán, Mexico City, Distrito Federal, Mexico; Instituto Nacional de Ciencias Médicas y Nutrición Salvador Zubirán, Mexico City, Distrito Federal, Mexico; Instituto Nacional de Ciencias Médicas y Nutrición Salvador Zubirán, Mexico City, Distrito Federal, Mexico; Instituto Nacional de Ciencias Médicas y Nutrición Salvador Zubirán, Mexico City, Distrito Federal, Mexico

## Abstract

Disparities in social determinants of health (SDH) can hinder older people’s health, regardless of their physical and functional status, for example, by limiting their access to health care. This study examines the association between SDH and the decision of older adults not to seek emergency medical care when needed, independent of their comorbidity status or intrinsic capacity. We conducted a secondary cross-sectional analysis derived from the 2021 National Survey on Health and Ageing in Mexico (ENASEM), which assesses ageing, disease impact, disability, and mortality among individuals aged ≥50 years. Weighted univariate and multivariate logistic regression models were performed to evaluate SDH as predictors of emergent healthcare-seeking behavior, adjusting for age, sex, intrinsic capacity, comorbidities, and functional status. Of 10,482 participants (median age: 71 years, IQR 65-77; 55.95% women), 560 (5.9%) had a serious health problem but did not seek medical care. For the main outcome, “being currently employed” was independently associated with higher odds of not seeking care (OR 1.64, 95% CI 1.02–2.63, p = 0.042), while “having social security” (OR 0.59, 95% CI 0.41–0.85, p = 0.004) and “benefiting from a social network” (OR 0.15, 95% CI 0.06–0.36, p < 0.0001) were found to be protective. Social programs, private transport, socioeconomic status, marital status, schooling, and rural vs urban location were not significantly associated. Our findings highlight the influence social factors may have on the access to emergency care, underscoring opportunities for advocacy for equity in the SDH.

